# The relationship of early life adversity and physiological synchrony within the therapeutic triad in horse-assisted therapy

**DOI:** 10.1007/s00702-025-02947-7

**Published:** 2025-05-27

**Authors:** Stella Wienhold, Larissa Bär, Zoe Ringleb, Victoria Zirpel, Annette Gomolla, Bernadette F. Denk, Nina Volkmer, Raphaela J. Gaertner, Elea S. C. Klink, Jens C. Pruessner

**Affiliations:** 1 Neuropsychology, Department of Psychology, University of Konstanz, Konstanz, Germany; 2 GREAT – German Research Center for Equine Assisted Therapy, Konstanz, Germany; 3 Centre for the Advanced Study of Collective Behavior, University of Konstanz, Konstanz, Germany

**Keywords:** Physiological synchrony, Autonomic nervous system, Inter-species synchronization, Horse-assisted therapy, Early life adversity, Trauma

## Abstract

In any therapeutic setting, the outcome depends in part on the therapeutic alliance, characterized by mutual understanding, empathy and trust among the participants. This also manifests through physiological synchronization (PS) processes involving breathing, heart and brain. This study examined the dynamics of heart rate variability (HRV) synchronization patterns during horse-assisted therapy. We explored the correlations between the therapist’s horse preference, levels of early life adversity (ELA), and PS relationships within and across dyads of participants, therapists, and therapy horses. Our sample of 42 female participants engaged in standardized horse-assisted therapy sessions facilitated by three riding therapists and four therapy horses. PS was operationalized through cross-wavelet power analyses across the different dyads. The results showed, that stronger HRV synchronization between the therapist and horse was associated with stronger HRV synchronization between the horse and participant, as well as stronger HRV synchronization between the therapist and participant. We found a correlation between ELA and HRV synchronization between participants and therapists, with individuals experiencing higher levels of ELA showing lower synchronization. However, this effect of ELA was not observed for HRV synchronization between participants and horses. Furthermore, we found a negative correlation between the riding therapist’s preference for a particular therapy horse and the HRV synchronization between the therapist and that horse. These findings contribute to a better understanding of the correlational dynamics in horse-human interactions and may have potential implications for optimizing therapeutic interventions in clinical settings.

**Supplementary Information:**

The online version contains supplementary material available at 10.1007/s00702-025-02947-7.

## Introduction

### Therapeutic alliance as the basis for healing experiences

At the heart of effective therapeutic interventions lies the therapeutic alliance (TA) – the nuanced connection built upon mutual understanding, empathy and trust between the therapist and patient that forms the foundation for therapeutic progress (Jackob and Hueß 2016; Rogers 1957). This foundation allows for the expression of thoughts and feelings and creates a safe space for patients to explore their vulnerability, which is an essential prerequisite for successful therapy. Furthermore, TA provides the basis from which therapists and patients can work together toward therapeutic goals. Considerable empirical evidence documents the positive impact of TA on treatment outcomes by contributing to more effective and lasting therapeutic results (Baier et al. 2020; Henley and Miller 2010; Huggett et al. 2022; Keller et al. 2010; Krupnick et al. 2006; Zilcha-Mano 2017).

### Therapeutic alliance between humans and horses

While TA has traditionally been described in human-to-human connections within therapeutic settings, the extension of the concept to horse-assisted therapy (HAT) introduces a unique dynamic that shifts the focus on the connection between human and horse, while the therapist remains in the background. In contrast to verbal articulation of thoughts and feelings, communication with horses relies primarily on non-verbal cues and creates a unique and deep connection between patient and horse that goes beyond words (Bivens et al. 2007; Scopa et al. 2019). In human-horse TA, participants enter a space where silent understanding and subtle gestures become the primary means of communication, creating an environment rich in unspoken dialog and mutual understanding (Smith et al. 2016; Wilson et al. 2017; Hameury and Rossetti 2022). As horses are non-judgmental by nature, they promote acceptance by being free from criticism and acting as a mirror by providing immediate and honest feedback (Gomolla 2020; Jones 2020).

### Components of therapeutic alliance

Koole and Tschacher (2016) introduced a comprehensive model of TA known as the In-Sync model, which emphasizes the role of interpersonal synchrony in fostering a strong therapeutic bond. In their model, they highlight several key components that contribute to the effectiveness of the TA across different levels of interaction dynamics: At level 1, the model underscores perceptual-motor processes that can be observed through phenomena such as movement synchrony and inter-brain coupling occurring on a phasic timescale. At level 2, the alliance manifests as a product of the interplay between the therapist’s and patient’s cognitive appraisals, beliefs and interpretations through complex cognition processes on a tonic timescale. Emotion regulation on level 3 is observable by both implicit and explicit emotion regulation processes over a chronic timescale.

Building upon their research, we propose that movement synchrony and inter-brain coupling represent distinct yet interconnected facets of the same underlying processes, operating at different hierarchical levels (Shamay-Tsoory et al. 2019). Physiological synchronization (PS), characterized by the harmonious alignment of processes of the autonomic nervous system (ANS) (Palumbo et al. 2017), can be assumed to happen in between movement synchrony and inter-brain coupling and represents another important pillar of synchronization. Thus, PS can be regarded as the link between neural dynamics and observable behavior within the therapeutic dyad, which is detectable by measuring synchronization of heart rate variability between the interaction partners (Beffara et al. 2016; Matusik et al. 2023; Tschacher and Meier 2020).

### Heart rate variability as a measure of the adaptability of the autonomic nervous system

Heart rate variability (HRV) is a measure quantifying the temporal variation between successive heartbeats and reflects the adaptability of the ANS in response to diverse physiological and environmental influences (Thayer and Lane 2000, 2009; Task Force of the European Society of Cardiology and the North American Society of Pacing and Electrophysiology 1996). Higher HRV is generally associated with increased adaptability and resilience of the ANS and therefore allows for assessments of general health and chronic stress levels in both humans and horses (Kemp and Quintana 2013; Kuwahara et al. 1996; Michels et al. 2013; Moon et al. 2013; Thayer et al. 2010).

The dynamics of cardiac activity include high- and low-frequency fluctuations, often referred to as high and low-frequency HRV (HF-HRV, LF-HRV). These variations are closely linked to the influence of the parasympathetic nervous system (PNS) and the sympathetic nervous system (SNS). The vagus nerve, a key component of the PNS originating in the brain stem, rapidly regulates the body’s organs, including the heart, through the release of acetylcholine. Therefore, high-frequency changes, characterized by oscillations of 0.15 Hz or less, are primarily associated with parasympathetic activity (Task Force of the European Society of Cardiology and the North American Society of Pacing and Electrophysiology 1996). In contrast, SNS innervation occurs more slowly through epinephrine and norepinephrine. Therefore, low-frequency changes with oscillations above 0.15 Hz are not exclusively indicative of sympathetic influence, as LF-HRV reflects a combination of both sympathetic and parasympathetic influences. At rest, LF-HRV is highly correlated with HF-HRV, suggesting significant parasympathetic involvement (Goldstein et al. 2011; Billman 2013). The measurement of both HF and LF variability can be achieved through various methods, with one common approach being the decomposition of the heart rate signal into different frequency bands, enabling the quantification of high- and low-frequency changes.

### Heart rate variability synchronization as a measure for the collective autonomic nervous system

When we refer to HRV synchronization, we define it as the coordinated interaction of heart rate patterns between individuals, implying a significant degree of physiological alignment or connection (Palumbo et al. 2017). When individuals experience synchronized HRV during interactions with others, it indicates a harmonious and correlated response of their ANS (Sharika et al. 2024). This phenomenon can serve as a dependent variable in empirical studies, allowing the investigation of shared physiological states and the effects of interpersonal dynamics in connection with overall well-being (Palumbo et al. 2017). Essentially, HRV synchronization provides insight into how individuals’ physiological responses are aligned during shared experiences or therapeutic interactions, expanding our understanding beyond the study of individual HRV (Timmons et al. 2015; Plonka et al. 2024).

#### Current research on heart rate variability synchronization

HRV synchronization has gained considerable attention in various fields, as it holds potential implications for understanding the complex dynamics of social interactions, stress resilience and overall well-being (daSilva and Wood 2024). Research in this area has traditionally focused on heart rate synchrony in familial relationships, such as between mothers and children or within couples (Feldman et al. 2011). HRV synchronization tends to manifest during physical touch, with additional factors such as gender dynamics and avoidance behaviors influencing the complex interplay of physiological states during social interactions (Chatel-Goldman et al. 2014). Other studies have investigated HRV synchronization in the context of choir singing and found increased synchronization during singing compared to resting periods (Müller et al. 2011; Ruiz-Blais et al. 2020).

#### HRV synchronization in horse-assisted therapy

Physiological synchronization processes extend not only to human interactions during psychotherapy (Scheidt et al. 2021; Ramseyer and Tschacher 2011; Tschacher and Meier 2020), but also to human-animal interactions, and therefore HRV synchronization may play a potential role within the therapeutic triad of therapy horse, riding therapist and participant during HAT. Previous research has demonstrated the positive effects of HAT on cardiac autonomic function (Baldwin et al. 2018; Ecker and Lykins 2019; García-Gómez et al. 2020; Gehrke et al. 2018; Park et al. 2021), but there is still little understanding of the nuanced interplay between ANS responses of humans and horses during therapeutic sessions. Understanding these interactions may provide insights into the mechanisms underlying emotional regulation (Lin et al. 2024), social bonding and therapeutic efficacy in HAT – providing evidence-based strategies to improve both client outcomes and animal welfare (Porges 2007; Fine 2010).

### The role of early life adversity

While PS in HAT is essential for understanding in-the-moment alignment, it is equally important to take into account how a person’s developmental history influences the overall tendency to synchronize. Early life experiences, particularly those involving adversity and trauma can disrupt physiological regulation and social engagement systems, thereby affecting both the capacity for and quality of interpersonal synchrony (Feldman 2007; Sigrist et al. 2021; Tell et al. 2018; Jankovic et al. 2021). Individuals who have experienced early life adversity (ELA), such as childhood abuse, neglect or family dysfunction, may face profound challenges in building trusting relationships and engaging in therapeutic processes. These adverse experiences can shape individuals’ attachment styles, affect regulation and interpersonal behaviors, and impact their ability to form and maintain meaningful connections with therapists (Jankovic et al. 2021; Meyer et al. 2016; Petrova et al. 2021; Sigrist et al. 2021; Tell et al. 2018).

From infancy, PS between caregivers and infants plays a crucial role in regulating the infant’s emotional and physiological states. Early interactions help infants develop essential skills for affect regulation, secure attachment and stress system development (Feldman et al. 2007). When these synchronizing processes are disrupted by ELA, such as through caregiver unavailability, neglect or abuse, infants may struggle to develop effective self-regulation skills and secure attachments (Shonkoff et al. 2012). Over time, this can result in a dysregulated stress response system and altered HRV patterns, which can manifest in different ways depending on the individual’s adaptive strategies. While some individuals with ELA exhibit reduced HRV reflecting impaired autonomic flexibility and heightened stress reactivity, others may present with elevated HRV, particularly in dissociative states, which can serve as a maladaptive coping mechanism to disengage from overwhelming stress (Sigrist et al. 2021). These divergent autonomic patterns highlight the complexity of physiological responses to ELA and suggest that dysregulated HRV does not always indicate a uniform stress-related impairment but may also reflect a state of autonomic disconnection. Difficulties in social interactions and therapeutic relationships are common consequences of these autonomic adaptations, as low HRV is associated with emotional rigidity and poor stress recovery, while paradoxically high HRV in dissociative states may reflect emotional detachment rather than true physiological flexibility (McEwen and Gianaros 2011; Sigrist et al. 2021; Thayer and Lane 2000, 2009). This dysregulation suggests that ELA can serve as a moderating factor in how effectively TA and PS occurs within the therapeutic dyad. While the In-Sync Model (Koole and Tschacher 2016) explains how synchronization fosters the therapeutic bond, ELA helps to explain why certain individuals may experiences impairments in achieving this synchrony. Therefore, understanding the impact of ELA on TA is essential for the development of interventions to meet the specific needs of affected individuals.

Unlike human interactions, where ELA can hinder trust and relationship building, we propose that TA with horses offers a safe space in which affected individuals can form deep and meaningful connections without the fear of judgment or rejection. Horses, being highly attuned to physiological signals provide immediate feedback, a nonjudgmental presence and opportunities for co-regulation (Gehrke et al. 2011). Through HAT, individuals with ELA can experience the benefits of co-regulation and attachment repair, which may later generalize to human relationships, including therapeutic settings.

### Research gap

The need for research within the therapeutic triad of HAT stems from its increasing popularity as an adjunct to conventional psychotherapy in the treatment of various psychiatric disorders and mental health issues (Beetz et al. 2012; Kendall et al. 2015; Nurenberg et al. 2015; Trotter et al. 2008). In contrast to conventional therapy settings, HAT utilizes the inherent social nature of horses and their ability to build deep relationships with humans (Anderson & Meintz 2016; Nurenberg et al. 2015). The therapy horse becomes an active participant, contributing to the therapeutic process through its non-judgmental presence, responsiveness and unique communicative skills (Carlsson et al. 2014; Gomolla 2020). Despite the growing evidence of its effectiveness (Borgi et al. 2016; Earles et al. 2015; Johnson et al. 2018; Palomar-Ciria and Bello 2023; Staudt and Cherry 2017; Ward et al. 2013), there is currently a lack of comprehensive understanding of the underlying mechanisms due to methodological shortcomings. Therefore, this study aims to fill this gap by investigating HRV synchronization dynamics within the therapeutic triad.

### Our hypotheses

Grounded in the fundamental understanding that the dynamics between the therapy horse and riding therapist are central to shaping the overall therapeutic environment, we intended to find out how these dyads influence other physiological interactions within the therapeutic triad. Specifically, we hypothesized that stronger HRV synchronization between riding therapist and therapy horse will significantly increase HRV synchronization in the other dyads of the triad (e.g., between participant and horse and between participant and therapist). This is based on the assumption that harmonious interactions between the therapy horse and riding therapist create a more comfortable and safer atmosphere for the participant, potentially enhancing the physiological alignment.

Based on prior research showing that heart rates synchronize more strongly during interactions with familiar individuals (Chatel-Goldman et al. 2014; Feldman et al. 2011; Naber et al. 2019), we hypothesized that HRV synchronization between the riding therapist and therapy horse might be strongest when the therapist is working with her favorite horse. This hypothesis aimed to explore whether a subjective sense of connectedness, potentially fostered by familiarity, could enhance physiological synchrony within human-horse interactions.

We further wanted to explore whether past experiences such as ELA can significantly influence an individual’s ability to form connections and synchronize with others within a therapeutic setting. Therefore, we postulated that participants with high ELA levels show significantly lower HRV synchronization within the therapeutic triad compared to participants with less or non-ELA. In conclusion, our research into HRV synchronization within the therapeutic triad is not just an academic pursuit, but a crucial step in exploring the potential of HAT and hopefully finding ways to improve therapeutic efficacy and create meaningful connections between humans and animals.

## Methods

The data collection relevant for this stud took place in the summer months (April-September) of 2019, 2020, and 2021, with face masks worn by both experimenters and participants during data collection in the summer of 2021 in adherence to COVID-19 guidelines. This study was preregistered at OSF-Repository (10.17605/OSF.IO/M7X94).

### Therapy horses

Four experienced therapy horses were involved, each with expertise in therapeutic work and different internal and external attributes, including variations in size, breed, and age. The team of horses consisted of an 8-year-old Connemara (Forest) and a 13-year-old Fjord gelding (Karlson), an 18-year-old Fjord (Jill) and a 19-year-old Criollo mare (Bonita). To ensure consistency across therapy sessions, the therapy horses were randomly assigned to participants and therapists. This approach was designed to control for potential biases related to therapist preference. In typical HAT settings, therapy horses are usually chosen based on the specific needs and therapy goals of the patient, as well as the suitability and temperament of the horse. However, in this study, random assignment was employed to maintain uniformity and avoid any influence of personal preferences on the HAT setting.

### Materials

During the HAT session, we used specialized equipment to ensure the safety and comfort of both the participants and the horses. This included a therapy halter, a lead rope, a therapy pad, and a belt with two handholds. The participants were also provided with brushes and helmets. The heart rates of participant, riding therapist and therapy horse were recorded using Polar chest belts with sensors (Polar 2022) connected to iPhones and iPads. The HRV Logger App (Altini 2013) was used on all three devices for data collection.

### Sample

Participants were recruited through flyers and posters in psychiatric institutions and practices around Constance. Eligible participants were physically healthy women aged between 18 and 45, who were non-smokers or consumed fewer than five cigarettes per day. Individuals with a history of illicit drug use, substance dependence or active alcohol abuse were excluded from the study. Participants taking psychiatric medications were not automatically excluded, but their medication status was recorded. They were also required to weigh less than 80 kg, in accordance with horse welfare guidelines to ensure the well-being of the therapy horse during sessions. They received 15€ as compensation per session. The sample comprised 56 healthy women in the control group and 36 women undergoing psychotherapeutic treatment in the patient group with various diagnoses such as depression, post-traumatic stress disorder, anxiety disorder, social phobia, psychosis, and adjustment disorder. The age of the participants included in the statistical analysis ranged from 19 to 45 years (*M =* 28.90, *SD =* 7.24).

### Therapists

The study involved three riding therapists, all female, whose age varied across the three years of data collection. Riding Therapist 1 (RT1) conducted data collection in 2019 and 2020 and was 26 years old in 2019. RT2 conducted data collection in 2020 and 2021 and was 21 years old in 2020. RT3 conducted data collection exclusively in 2021 and was 22 years old at that time. All therapists were licensed riding therapists (Institut für pferdegestützte Therapie, IPTh, Konstanz, Germany) with at least one year of experience in HAT. To ensure consistency across sessions, they adhered to a standardized therapeutic approach, minimizing therapist-related variability in HRV synchronization by maintaining consistency in verbal and nonverbal interaction with participants. While their primary role was to facilitate equine interactions rather than provide direct psychological intervention, their level of engagement varied depending on participant needs.

### Questionnaires

Prior to the actual HAT sessions, participants completed an online screening questionnaire about age, weight, gender, and mental and physical health. ELA was assessed using the Childhood Trauma Questionnaire – CTQ (Wingenfeld et al. 2010), a widely accepted instrument for assessing various forms of childhood maltreatment. This questionnaire includes 28 items in the five subscales Emotional, Physical and Sexual Abuse as well as Emotional and Physical Neglect.

Before the actual HAT segments, participants provided informed consent and completed additional questionnaires, including a 0–10 visual analog scale (VAS) assessing acute stress levels. Chronic stress levels were evaluated in the patient group during the second session using the Perceived Stress Scale – PSS (Cohen et al. 1994). This widely used measure captures the extent to which situations in life were perceived as stressful over the past month. The participants in the patient group further completed the Shutdown Dissociation Scale – Shut-D questionnaire (Schalinski et al. 2015) during the third session. Additionally, in both the first and final sessions, participants in the patient group rated their symptoms, perceived stress severity and the impact on personal and professional lives using a VAS of 0–10. After each HAT session, participants in both groups provided detailed feedback on their overall experiences and interaction quality.

### Design

**Fig. 1:**
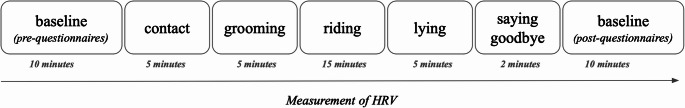
Timeline of data collection in minutes Note. The timeline presented illustrates the sequence and duration of activities involved in the data collection process, specifically in minutes

Participants who passed the online screening and met the inclusion criteria were invited to the data collection sessions at the test site, a rural farm at the outskirts of a village near Singen, Germany. These appointments took place either at 8:45 a.m. or 10:30 a.m. and lasted 60 minutes. The participants in the control group took part in one standardized HAT session, while the patient group took part in four sessions. In the beginning, the participants were informed about the aims of the study and possible risks. After giving consent, they put on the Polar chest belt with sensor (Polar 2022). The riding therapist then started the recording of the heart rates using a paired iPhone or iPad. Next, participants completed questionnaires for about ten minutes. This was followed by the five distinct parts of patient – horse interaction, namely contact, grooming, riding, lying on the horse, and farewell. The first, a five-minute contact session with the horse, included the participants interacting with and stroking the horse. The riding therapist then instructed the participants in grooming the horse for five minutes. Afterwards, the riding session followed, where the horse was fitted with a therapy pad and a riding belt and taken to the riding arena for a fifteen-minute riding session, which included two short trotting sequences. After riding, the horse was brought to a halt in the middle of the riding arena and the participant was instructed to lie on the horse’s back in a prone position for five minutes. The horse was then returned to the therapy box and the participant was allowed to say goodbye to the horse before taking part in a ten-minute questionnaire session. In the end, the HRV chest belt was removed and the participants received €15 as compensation. An overview of the procedure is shown in Fig. 1. The measurement of heart rate was similar for participants, riding therapists, and therapy horses.

### Data analysis

#### Data cleaning

Due to erroneous data collection, we obtained a considerable amount of incomplete heart rate recordings for therapists. Therefore, only 42 participant-horse-therapist triads could be included in the subsequent statistical analysis, consisting of 9 participants in the control group and 33 in the patient group. This significant amount of missing data of therapists can be explained by the initial focus of recordings from participants and therapy horses, resulting in frequently incomplete riding therapist data.

#### Data processing

Raw RR-interval data were processed using the statistics program R (R Core Team 2020), employing lab-internal R-scripts, which were partially adapted to suit the specific structure of our data. Initial preprocessing included identifying and correcting double entries, outliers and missing data points. Outliers were defined based on RR-interval inspection and applying a threshold ranging from 20 to 50%, ensuring realistic physiological boundaries. Missing data segments shorter than two consecutive seconds were interpolated by using the values immediately preceding and following the gaps. Longer segments were interpolated using longer pre- and post-sections of real data as best estimate. Despite using a recording device specifically designed for horses (‘Polar Equine heat rate riding belt’), the heart rate recordings from horses were more error prone than those of the humans, perhaps because of interference with the therapy pad, the thick skin of the horses, or frequent shifting of the belt, such that our standard preprocessing scripts for artifact detection and removal could not be used routinely in the equine data sets. Consequently, we applied specific artifact recognition and interpolation analyses on multiple horse data sets to effectively eliminate occurring artifacts. For this purpose, we visually inspected all individual RR data points of the horses and applied a semi-manual algorithm to remove artifacts for individual sections and finally interpolated missing values using our standard scripts. Following these corrections, all artifact-free datasets underwent our standard processing pipeline including R packages RHRV (Rodrígues-Liñares et al. 2011), parsedate (Csardi and Torvalds 2019), haven (Wickham and Miller 2020), foreign (R Core Team 2020a), WaveletComp (Roesch and Schmidbauer 2018), dplyr (Wickham et al. 2022) and lubridate (Grolemund and Wickham 2011) for calculating and extracting HRV parameters of relevant data sections.

### Cross-wavelet power analyses

The assessment of HRV synchronization involves analyzing the temporal coherence of cardiac oscillations between two interacting systems. While numerous methods exist for this purpose (Denk et al. 2024), we applied cross-wavelet power analyses, a statistical technique used in signal processing and time-frequency analysis to assess the coherence or synchronization between two signals over time (Grinsted et al. 2004; Maraun and Kurths 2004). Cross-wavelet power analysis assesses the power of synchronization of two time series over time and across frequency bands (Torrence and Compo 1998). This method allows to identify regions over time and across frequency bands where the oscillations of two signals are synchronized and is therefore particularly useful for studying complex systems where multiple interacting factors influence physiological outcomes.

In the specific context of HRV synchronization, cross-wavelet power analysis offers significant methodological advantages, allowing us to identify specific frequency bands where heart rate patterns of interaction partners align most closely over time. Notwithstanding Heisenberg’s uncertainty principle, which emphasizes the inherent trade-off between temporal and frequency resolution in this type of analysis (Heisenberg 1927), this method can enhance our understanding of physiological synchronization with more fine-grained temporal and frequency resolution (Denk et al. 2024).

Therapeutic settings pose a challenge to any form of standardized analyses, since timing and length of the individual therapy segments in practice inevitably vary, resulting in fluctuations across sessions over several minutes. This complicates the analysis process, as heart rate data must be divided into equally sized intervals across all dyads in the groups, which only then can be submitted for further analyses. As a result, and to ensure compatibility across sessions and dyads, we selected four consecutive one-minute intervals during the segment lying (L) throughout the sessions for all observations, given its strong association with parasympathetic dominance and stable physiological conditions.

Following data processing, initial wavelet analyses were conducted using the RHRV package (Rodrígues-Liñares et al. 2011) for data interpolation, frequency analysis and power band calculations. Subsequently, cross-wavelet power analyses were performed using the WaveletComp package (Roesch and Schmidbauer 2018), allowing precise specification of analysis parameters regarding time and frequency resolution.

To leverage the high-resolution capability of cross-wavelet power analyses, HRV was categorized into four frequency bands: upper high frequency (UHF), lower high frequency (LHF), upper low frequency (ULF) and lower low frequency (LLF). Given the expected parasympathetic synchronization during the “lying” therapy segment, our analyses specifically concentrated on the high-frequency bands (UHF & LHF), typically referred to as ‘HF’ in standard HRV analyses based on Fast-Fourier transform. Finally, we z-transformed the cross-wavelet power data to normalize the distribution, allowing for more accurate comparisons and statistical analysis across sessions and dyads.

### Statistical analysis

We employed a strictly hierarchical structure of cross-wavelet power data with 10-second intervals nested within minutes, minutes nested within therapy segments, therapy segments nested within HAT sessions, and HAT sessions further nested within participants or riding therapists. We then employed multi-level models with repeated measurements (RM-MLM). All statistical analyses were carried out using R, along with the packages nlme (R Core Team 2020b) and lme4 (Bates et al. 2015, 2022).

We chose maximum likelihood estimation due to its robustness and unbiased estimates in large samples. For inferential statistical testing, various RM-MLMs were established and compared. The analyses began with a null model (Model 0) that lacked adjustments for predictor variables. Subsequent models were introduced sequentially, incorporating random intercepts, random slopes and the predictor variables fitting the study design (Session, Minute, Interval, Frequency, and their interactions). For further information on RM-MLM, please refer to Bryk and Raudenbush (1992), Hox et al. (2017) and Snijders and Bosker (2012).

As HRV is known to be influenced by multiple physiological and psychological factors (Sammito et al. 2024; Kemp et al. 2010), we systematically included age, body mass index (BMI), medication usage, experiences with horses and mental health group membership (whether participants belonged to the control or patient group) as covariates in initial models. These variables were tested for their impact on model fit to ensure that the observed effects were not biased by individual differences while minimizing potential confounding variability.

In addition, we calculated mean baseline HRV for both participants and riding therapists using the Root Mean Square of Successive Differences (RMSSD) over the four-minute segment and included these as covariates in the respective models. Baseline HRV was retained if it significantly improved model fit.

Once the best-fitted model was identified, the influence of proposed factors such as Favorite Therapy Horse, HF-HRV Synchronization between Therapy Horse and Riding Therapist as well as CTQ subscales was evaluated by adding these as independent variables and comparing the respective models. Analyses of variance (ANOVAs) were conducted with a predetermined alpha level of 5% to determine which model better fitted the data.

While ELA was analyzed continuously via CTQ subscales in all statistical models, we additionally grouped total scores into categories for illustrative purposes (e.g. in Figs. 4 and 5). These groupings were not used for hypothesis testing and were applied for visualization only; they do not reflect standardized clinical thresholds but were based on approximate score ranges informed by the distribution of the sample.

#### Assumption checks

We performed a series of diagnostic test to evaluate the underlying assumptions of the statistical analyses. These assessments included examining the normality of residuals using QQ plots, evaluating the independence of residuals through scatterplots, and assessing homoscedasticity via residual plots (Fox 2008). Furthermore, potential outliers were examined, and the absence of multicollinearity was confirmed by studying correlations between predictors (Neter et al. 1989). These checks ensure the robustness and validity of the analyses. Given the robustness of ANOVAs and RM-MLMs to deviations from normality, exceptions where a strict adherence to normal distribution assumptions was not met were still considered in the statistical analysis (Blanca Mena et al. 2017; Quené and Van den Bergh 2004; Schmider et al. 2010).

## Results

Our analyses focused on the extracted cross-wavelet power (CWP) values across the frequency bands from the cross-wavelet power plots. This approach is comparable to traditional high and low frequency band analysis using Fast Fourier transform, except that we here benefit from higher temporal and frequency resolution (see Methods). Readers who would like an introduction to CWP analysis and its applications are recommended to consult Grindsted et al. (2004), Torrence and Compo (1998) and Paluš (2014).

**Fig. 2:**
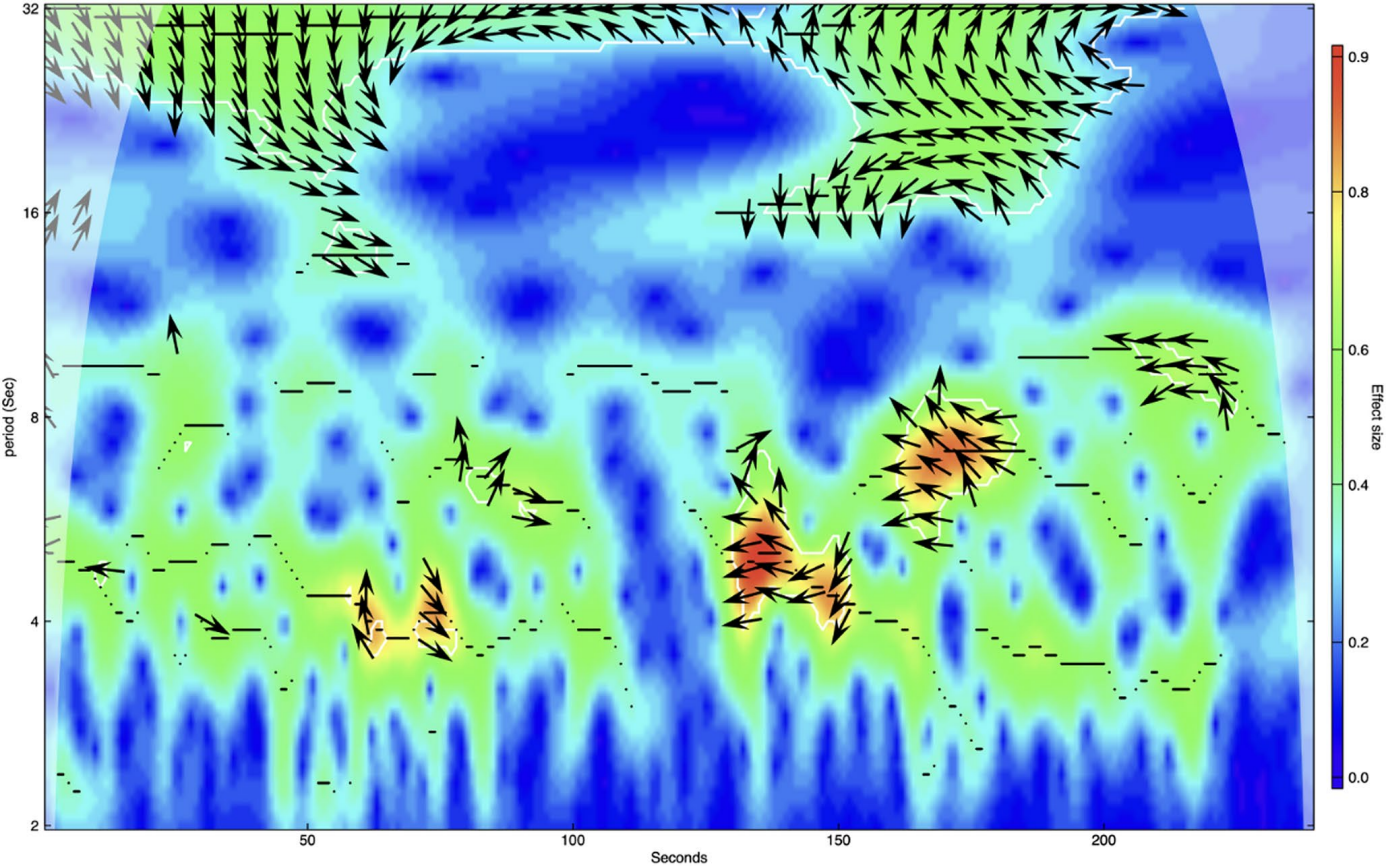
Cross-wavelet power analysis of HRV synchronization: therapy horse and participant during a four-minute segment in an initial HAT session Note. The figure displays a cross-wavelet power analysis of HRV synchronization between the therapy horse and the participant during a four-minute segment in an initial HAT session. The color scale represents the effect size, with warmer colors (red and orange) indicating higher synchronization levels and cooler colors (blue and green) indicating lower synchronization levels. The arrows represent the phase relationship between the HRV signals of the horse and the participant, with the direction of the arrows showing the phase angle. The x-axis represents the time in seconds, while the y-axis represents the period in seconds (the duration of each cycle in the HRV signals, which is inversely related to frequency by the relationship Period = 1/Frequency)

To illustrate how we measured PS, Fig. 2 shows a cross-wavelet power plot of HRV synchronization patterns between a therapy horse and a participant during the “lying” segment of an initial HAT session. This plot captures both the timing and frequency of HRV synchrony over a four-minute period. The x-axis represents time in seconds, while the y-axis indicates the wavelet period (in seconds), which is inversely related to frequency (e.g. periods < 6.67 correspond to HF-HRV, typically associated with parasympathetic activity). Color intensity reflects synchronization strength with warmer colors (e.g. red, orange) denoting higher cross-wavelet power, i.e. stronger HRV alignment within the dyad. White contour lines highlight statistically significant regions of HRV synchrony (*p* <.05), and the arrows indicate the phase relationship between signals: Rightward arrows denote in-phase synchrony, leftward arrows indicate anti-phase synchrony, while the deviation from the horizontal line reveals which time series leads or follows, providing insights into leader-follower dynamics (see Roesch and Schmidbauer 2018).

In this example, significant HRV synchronization is seen especially within periods between 4 and 16, indicating coordinated activity across both HF-HRV and LF-HRV bands, but most notably in the HF range. This suggests that during this calm phase of the session, both therapy horse and participant were engaged in a shared, relaxed physiological state – likely driven by PNS activity and attunement (e.g. vagal coordination). Synchrony in the LF range may reflect broader autonomic co-regulation involving both sympathetic and parasympathetic branches.

Based on the hierarchical structure of our data, we used multi-level models with repeated measurements and identified which model structure fitted the data best. Models incorporated random intercepts, random slopes and predictor variables such as Session, Minute, Interval (10 s), Frequency and their interactions. The influence of proposed factors like Favorite Therapy Horse, HF-HRV Synchronization between Therapy Horse and Riding Therapist and ELA (measured with CTQ subscales) was then evaluated by adding them as predictor variables and comparing the respective model fits through ANOVAS, with a predetermined alpha level of 5%.

### Correlation of horse-therapist HF-HRV synchronization and horse-participant HF-HRV synchronization

The reference model predicting HF-HRV synchronization between therapy horse and participant, which included random intercepts, random slopes, covariates (BMI, Baseline HRV and Mental Health of participant) and design variables (Minute, Interval, Frequency and the interactions), demonstrated a significant enhancement in model fit upon adding the predictor variable HF-HRV Synchronization between Therapy Horse and Riding Therapist, χ2 = 1770.83, *p* <.01. This impact indicates a positive association, with an estimated value of 0.54 (CI: 0.52–0.57, *p* <.01). The final model predicting horse-participant HF-HRV synchronization demonstrated a marginal R^2^ of 0.38 and a conditional R^2^ of 0.52, indicating that the fixed effects explained 38% of the variance, while the full model, including the random effects, accounted for 52%. Table 1 contains model parameters and related statistical measures of the best-fit model predicting HF-HRV synchronization between therapy horse and participant. Further statistics on the model comparisons and parameters are available in Table S1 in the supplementary section.

### Correlation of horse-therapist HF-HRV synchronization and therapist-participant HF-HRV synchronization

Adding the predictor variable HF-HRV Synchronization between Therapy Horse and Riding Therapist to the reference model, which contained random intercepts, random slopes, covariates (Age and Baseline HRV of riding therapists), design variables (Minute, Interval, Frequency and the interactions), significantly contributed to model fit improvement, χ2 = 757.87, *p* <.01. This association appears to be positive, with an estimate of 0.36 (CI: 0.33 to 0.38, *p* <.01). The final model predicting HF-HRV synchronization between the riding therapist and participant showed a marginal R^2^ of 0.32 and a conditional R^2^ of 0.39, indicating that 32% of the variance was explained by fixed effects and 39% by the full model including random effects. Table 2 provides model parameters and related statistical measures of the best-fit model predicting HF-HRV synchronization between riding therapist and participant. Further statistics on the model comparisons and parameters are available in Table S2 in the supplementary section.

### Riding therapists and their favorite therapy horse

**Fig. 3:**
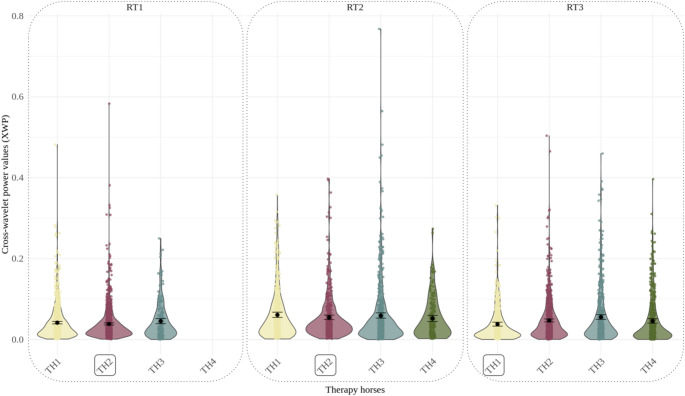
HF-HRV synchronization of therapy horses and riding therapists Note. This figure illustrates HF-HRV synchronization via cross-wavelet power values between therapy horses (TH1 - TH4) and riding therapists (RT1 - RT3). Violin plots show data distribution, with jittered points and black dots indicating mean values. Error bars represent 95% confidence intervals from bootstrapping. The high density of data points can be explained by obtaining one value per every 10 s for each session in our analysis. The black boxes mark the favorite therapy horse for each riding therapist, highlighting the cross-wavelet power value distributions specific to these preferred horses

The reference model predicting HF-HRV synchronization between the therapy horse and riding therapist, including random intercepts, random slopes, the covariate Baseline HRV of riding therapist, design variables (Minute, Interval, Frequency and the interaction Minute x Interval) and the predictor variable Therapy Horse, was improved by the addition of the predictor variable Favorite Therapy Horse, χ2 = 4.28, *p* <.05. The preference for a specific therapy horse showed a negative influence on HF-HRV synchronization between therapy horse and riding therapist, with an estimate of -0.07 (CI: -0.11 - -0.00, *p* <.01). The model predicting HF-HRV synchronization between therapy horse and riding therapist explained a modest proportion of variance with a marginal R^2^ of 0.23 and a conditional R^2^ of 0.25, suggesting that 23% of the variance was explained by fixed effects and 25% by the full model including random effects. Table 3 contains model parameters and related statistical measures of the best-fit model predicting HF-HRV synchronization between therapy horse and riding therapist. As shown in Fig. 3, HF-HRV synchronization patterns varied substantially across dyads and riding therapists (RT1 - RT3) did not consistently show higher physiological synchronization with their preferred horses (TH1 – TH4). Full model parameters are reported in Table 3, with supplementary model comparisons available in Table S3.

**Table 1:**
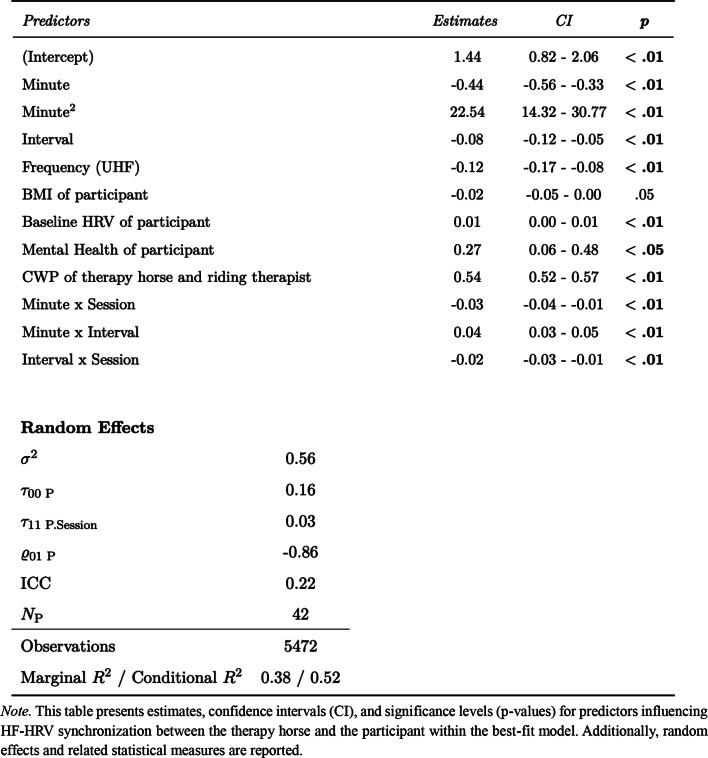
Model parameters and goodness of fit for best-fit model predicting HF-HRV synchronization between therapy horse and participant

**Table 2:**
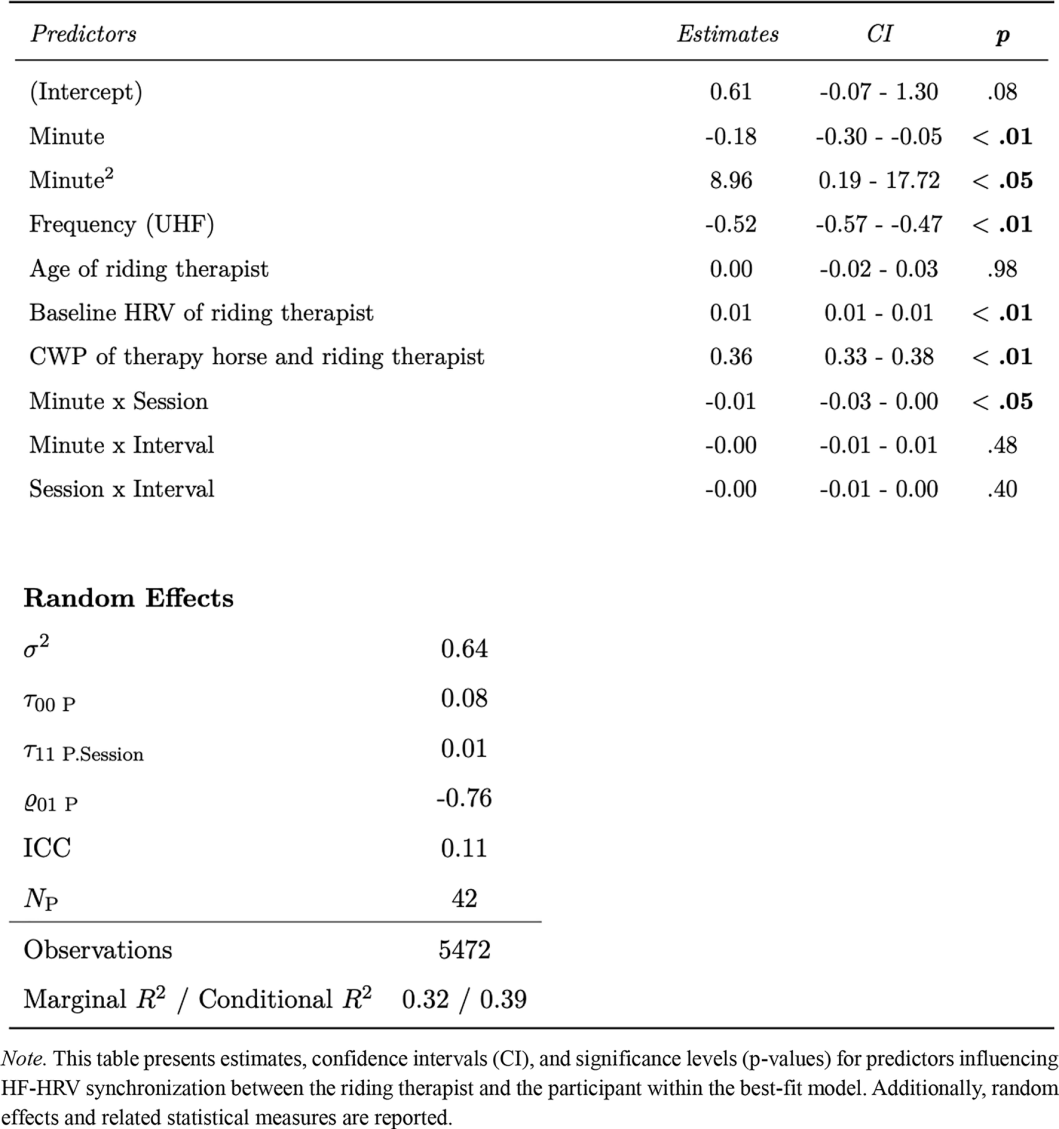
Model parameters and goodness of fit for best-fit model predicting HF-HRV synchronization between riding therapist and participant

**Table 3:**
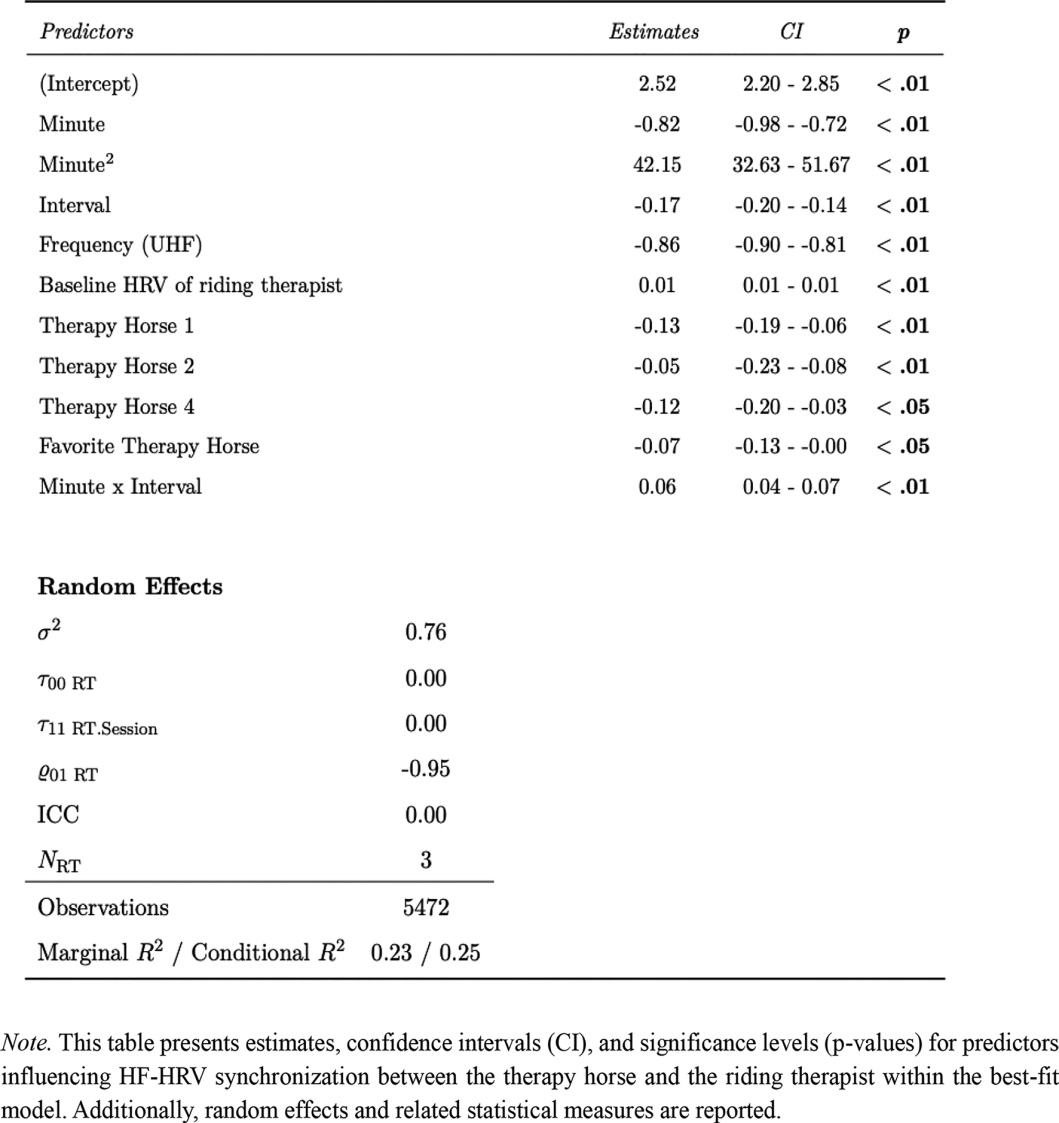
Model parameters and goodness of fit for best-fit model predicting HF-HRV synchronization between therapy horse and riding therapist

### Correlation of early life adversity and horse-participant HF-HRV synchronization

**Fig. 4:**
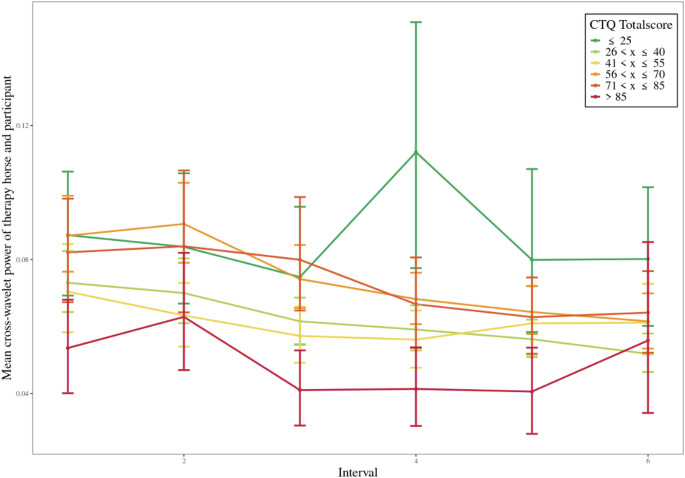
Impact of childhood trauma severity on mean cross-wavelet power between therapy horse and participant. Mean cross-wavelet power therapy horse and participant across intervals (10 s), stratified by Childhood Trauma Questionnaire (CTQ) total scores. Participants were grouped based on their CTQ total scores into the following categories: x ≤ 25 (green), 26 < x ≤ 40 (yellow-green), 41 < x ≤ 55 (yellow), 56 < x ≤ 70 (orange), 71 < x ≤ 85 (red-orange), and > 85 (red). Each point represents the mean value for the group in a given interval, and the lines connect the means across intervals. Error bars represent 95% confidence intervals calculated using bootstrap resampling. The color gradient from green to red visually represents increasing CTQ total scores, indicating higher levels of reported childhood trauma

We assessed ELA with the Childhood Trauma Questionnaire (CTQ – Wingenfeld 2010) and added the subscales (CTQ Totalscore, Emotional Abuse, Emotional Neglect, Physical Abuse, Physical Neglect and Sexual Abuse) as predictor variables in the respective reference models to explore their influence on HF-HRV synchronization. The models incorporated random intercepts, random slopes, covariates (BMI, Baseline HRV and mental health of participant), design variables (Minute, Interval, Frequency and the interactions Minute x Session, Minute x Interval and Interval x Session), and the predictor variable HF-HRV synchronization between Therapy Horse and Riding Therapist (M1). The inclusion of some of the CTQ predictor variables to the reference model predicting HF-HRV synchronization between therapy horse and participant did yield a discernible improvement in respective model fits. For example, CTQ Totalscore (χ^2^ = 4.50, *p* <.05), Emotional Abuse (χ^2^ = 4.99, *p* <.05), Emotional Neglect (χ^2^ = 4.98, *p* <.05) and Physical Neglect (χ^2^ = 3.88, *p* <.05) showed statistically significant improvements. However, Physical Abuse (χ^2^ = 1.22, *p* =.27) and Sexual Abuse (χ^2^ = 0.47, *p* =.49) did not lead to significant improvements. All the CTQ predictor variables that were included in the respective models showed statistical significance, with estimates ranging from 0.00 to 0.02, indicating a slightly positive effect on the dependent variable. Figure 4 illustrates the mean cross-wavelet power between therapy horse and participant across 10-second intervals, categorized by CTQ total scores, providing a visual representation of the potential relationship between childhood trauma severity and HF-HRV synchronization during therapy sessions. Further statistics on the model comparisons and parameters are available in Table S4 in the supplementary section.

### Correlation of early life adversity on therapist-participant HF-HRV synchronization

However, ELA showed a negative correlation with the HF-HRV synchronization between riding therapist and participant. The inclusion of the CTQ predictor variables (CTQ Totalscore, Emotional Abuse, Emotional Neglect, Physical Abuse and Sexual Abuse) to the reference model predicting HF-HRV synchronization between riding therapist and participant (M2) resulted in a noticeable improvement in model fit. The reference model included random intercepts, random slopes, covariates (age and Baseline HRV of riding therapist), design variables (Minute, Frequency and the interactions Minute x Session, Minute x Interval and Interval x Session), and the predictor variable HF-HRV Synchronization between Therapy Horse and Riding Therapist.

**Fig. 5:**
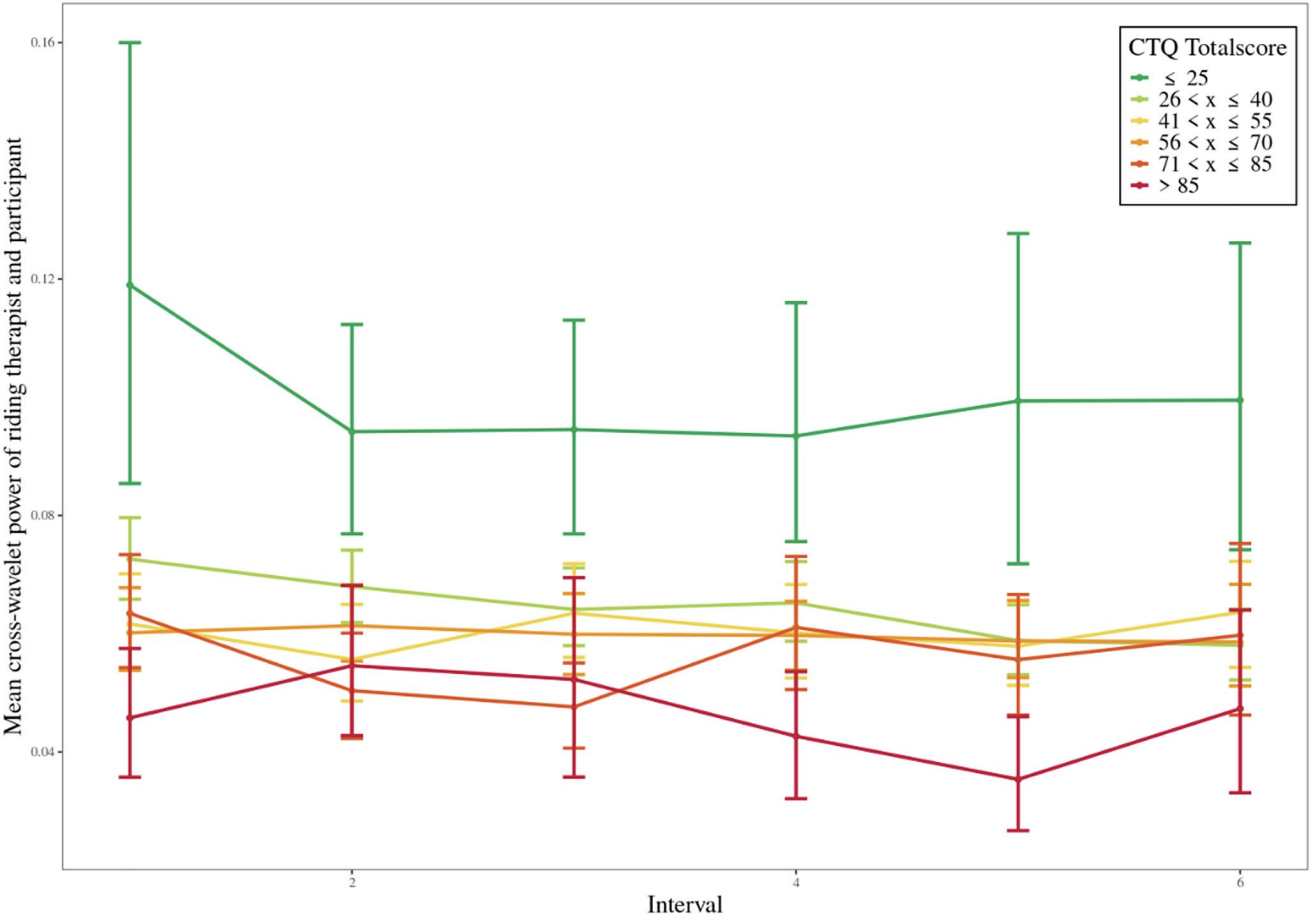
Impact of childhood trauma severity on mean cross-wavelet power between riding therapist and participant *Note.* Mean cross-wavelet power therapy horse and participant across intervals (10 s), stratified by Childhood Trauma Questionnaire (CTQ) total scores. Participants were grouped based on their CTQ total scores into the following categories: x ≤ 25 (green), 26 < x ≤ 40 (yellow-green), 41 < x ≤ 55 (yellow), 56 < x ≤ 70 (orange), 71 < x ≤ 85 (red-orange), and > 85 (red). Each point represents the mean value for the group in a given interval, and the lines connect the means across intervals. Error bars represent 95% confidence intervals calculated using bootstrap resampling. The color gradient from green to red visually represents increasing CTQ total scores, indicating higher levels of reported childhood trauma

The CTQ subscales CTQ Totalscore (χ^2^ = 5.69, *p* <.05), Emotional Abuse (χ² = 4.64, *p* <.01), Emotional Neglect (χ² = 6.67, *p* <.01), Physical Abuse (χ² = 4.72, *p* <.05) and Sexual Abuse (χ² = 5.08, *p* <.01) significantly improved the model. In contrast, the subscales Physical Neglect and Sexual Abuse did not contribute significantly to model fit (χ² = 1.29, *p* =.25, χ² = 2.71, *p* =.1). The estimates for the statistically significant CTQ subscales ranged from − 0.00 to -0.03, indicating a small negative effect on the dependent variable. Figure 5 illustrates the mean cross-wavelet power between riding therapist and participant across 10-second intervals, categorized by CTQ total scores, providing a visual representation of the potential relationship between childhood trauma severity and HF-HRV synchronization during therapy sessions. Further statistics on the model comparisons and parameters are available in Table S5 in the supplementary section.

## Discussion

This study represents the first in the application of cross-wavelet power analysis to study physiological synchrony between humans and horses during HAT sessions and offers new insights into the complex dynamics of physiological responses of the ANS within the therapeutic triad.

### The interaction of HF-HRV synchronization processes within the therapeutic triad

This study shows a robust positive correlation: high HR-HRV synchronization between the riding therapist and the therapy horse is significantly associated with high HR-HRV synchronization between the therapy horse and the participant.

Further investigations into the relationship between the HR-HRV synchronization between the therapy horse and the riding therapist and the subsequent HR-HRV synchronization with the participant yielded significant results. High HR-HRV synchronization is not only positively associated with high HR-HRV synchronization between the therapy horse and the participant, but also with high HR-HRV synchronization between the riding therapist and the participant.

The model predicting participant-horse synchronization demonstrated substantial explanatory power, with a marginal R^2^ of 0.38 and a conditional R^2^ of 0.52, indicating that half of the variance was accounted for when both fixed and random effects were included. In contrast, the model predicting therapist-participant synchronization explained less variance overall (R^2^ = 0.32/ 0.39), suggesting that physiological alignment in this dyad may be influenced by additional individual or interpersonal variables not captured in the current model.

These results emphasize the crucial role of the relationship between riding therapist and therapy horse in shaping the overall PS within the HAT sessions. However, it is important to note that these results are correlative, and no direct causal relationship can be inferred. The quality and coherence of the interaction between the riding therapist and the therapy horse could be a key factor influencing the overall dynamics of the therapeutic triad. Cultivating a strong and coordinated relationship between these key players may therefore have far-reaching implications for the effectiveness and impact of HAT sessions, but the exact causal mechanisms remain to be investigated.

This discovery aligns with the theoretical framework of HAT (Gomolla 2020) and incorporates insights from Attachment Theory. According to this theory, early interactions with caregivers significantly impact lifelong relationships and health outcomes (Bowlby 1965, 1969, 1971). In the context of HAT, the therapy horse acts as an important bridge for emotional and physiological cues between the riding therapist and the participant, embodying the principles of secure attachment (Bowlby 1969). The horse’s inherent sensitivity and responsiveness serve as powerful mediators of emotional states (Carlsson et al. 2014) and facilitate the transfer of physiological coherence from the therapy horse to the riding therapist and, subsequently, to the participant. This synchronization transfer is shown to be a critical element in creating an environment conducive to therapeutic progress, aligning with the broader understanding of secure attachment as a foundational element for well-being and effective interpersonal dynamics (Bowlby 1969).

Although the horse’s sensitivity may contribute to the promotion of physiological coherence, this coherence reflects correlations rather than direct effects. The observed synchronization transmission is part of a complex system of interconnected dyads in which each dyad reciprocally influences the others. Therefore, physiological coherence within the therapeutic triad should not be understood as a linear cause-effect relationship, but as an emergent property of mutual influence between all constituent dyads. For example, the degree of HF-HRV synchronization between the riding therapist and the therapy horse can not only reflect their interaction but also influence the subsequent synchronization between the therapy horse and the participant as well as between the riding therapist and the participant. Conversely, the HF-HRV synchronization between the riding therapist and the participant can also influence the HF-HRV synchronization processes of the other two dyads, and so on. This dynamic interaction suggests a network of interconnected physiological responses, where changes in one dyad ripple throughout the entire triad. Thus, rather than a linear causal relationship, the physiological coherence observed within the therapeutic triad is better understood as a result of the complex and mutually reinforcing relationships among its constituent parts.

### The therapy horse – riding therapist dyad

Through the incorporation of random intercepts and slopes in the model for predicting HF-HRV synchronization between therapy horse and riding therapist, we found significant variations in synchronization within and across dyads. These variations can be attributed to individual factors such as baseline heart rate, respiratory patterns, and overall cardiovascular health as well as individual stress levels, anxiety, and overall comfort during sessions.

Despite our efforts to standardize the HAT sessions and minimize interference, some environmental factors such as ambient noise and unexpected distractions could not be fully controlled, even at our rural farm. While this presents challenges for scientific rigor, it also enhances the generalizability of our findings, as our study environment more closely reflects the realities of everyday HAT setting than a controlled laboratory would.

The therapy session’s specific activities are crucial, such as the stationary “lying” segment during which the horse and riding therapist remain still for five minutes. The participant lies on the horse’s back during this segment, resulting in increased physical proximity which intensifies synchronization between the participant and the therapy horse. This heightened connection is associated with increased synchronization between the therapy horse and the riding therapist and creates a complex interplay of cues, responses, and dynamic interactions.

Including horse preferences of the therapist, as indicated by the ‘Favorite Therapy Horse’ predictor, significantly improved model fit and thus helped explain variance in HF-HRV synchronization between therapy horse and riding therapist. Surprisingly, results showed a negative correlation between horse preference of the therapist and HF-HRV synchronization within this dyad, contradicting the initial hypothesis. The preference for a specific therapy horse was on average associated with a lower HF-HRV synchronization between therapist and horse. This unexpected finding challenges the assumed positive influence of horse preference on the therapeutic alliance during HAT sessions and highlights the complexity of horse-human interactions in the therapeutic context.

Although the therapy horses were randomly assigned to control for potential biases related to therapist preference, this standardization may limit the generalizability of the findings to real-world HAT settings. In practice, therapy horses are usually selected based on various needs. These factors, which influence the dynamics of physiological synchronization in natural therapeutic environments, were not considered under the controlled conditions of this study. As a result, the results may not fully capture the nuances of horse-human interactions that occur in more personalized therapeutic environments.

One possible explanation for the unexpected negative effect of horse preference on HF-HRV synchronization could be related to the dynamics within the therapeutic relationship. It is conceivable that therapists with a strong preference for a particular horse may consciously or unconsciously bring certain expectations or biases into the interaction, potentially altering their behavior or communication style during sessions. Similarly, the horse itself may pick up on these subtle cues and respond in ways that unintentionally disrupt synchronization with the therapist. Given that horses are highly sensitive to human emotions, they may detect tension, discomfort, or incongruence in the interaction, which could affect their level of engagement or receptiveness during therapy.

Another potential explanation is that familiarity may reduce the need for active synchronization. When a therapist and horse have developed a strong bond over time, the need for conscious, moment-to-moment physiological alignment may decrease. In this sense, HRV synchronization could be seen as an adaptive process - one that facilitates alignment and attunement in newer or less familiar relationships but becomes less essential as the relationship matures and operates on a more implicit, automatic level. Here, lower HF-HRV synchronization may not indicate a weaker therapeutic alliance but rather an efficient interaction, where active synchronization is less critical to maintain attunement. This perspective suggests that HF-HRV synchronization plays a more critical role when there is a need to establish rapport, but as the relationship stabilizes, the reliance on this form of physiological alignment may diminish. As the therapy horse and the riding therapist are less physiologically synchronized to each other, they can respond better to the environment and therefore to the therapeutic setting. As a secure basis within the triad, they can perceive stimuli in the therapeutic triad independently of each other and benefit from the fact that they have a double set of eyes and ears (DaSilva & Wood 2024; Gomes & Semin, 2020). Further exploration of the mechanisms underlying this negative effect could offer valuable insights into the intricate interplay between psychological and physiological factors within HAT. Overall, the model predicting therapist-horse HF-HRV synchronization explained a smaller proportion of variance (R^2^ = 0.23/ 0.25), suggesting that additional contextual, relational, or affective variables may play a substantial role in shaping this dyadic physiological alignment.

### The correlation of early life adversity and HF-HRV synchronization

The exploration of the influence of ELA on HF-HRV synchronization patterns showed a notable distinction. We observed a significant negative association between ELA and HF-HRV synchronization in the participant’s interaction with the riding therapist. In contrast, this negative correlation was not observed in the participant’s synchronization with the therapy horse. This suggests that individuals with pronounced ELA exhibited lower levels of physiological synchronization with riding therapists, but this did not affect their synchronization with therapy horses during HAT sessions. The diminished capacity for physiological synchronization in individuals with ELA may stem from challenges in forming secure attachments and trust with other humans due to inconsistent or unreliable caregivers. Therapy horses, being non-human entities, in contrast, might offer interactions perceived as non-judgmental, free from complex social dynamics, making them more approachable also for individuals with ELA. The intuitive and sensitive nature of horses fosters a sense of safety and comfort, facilitating immediate physiological connection, even in individuals with pronounced ELA. The human-horse bond adds a nuanced layer, with horses mirroring and responding to human emotions and physiological states, creating a unique avenue for attunement and connection, less influenced by past traumatic experiences. This aligns with the emphasis of Attachment Theory on secure bonds fostering emotional well-being and resilience (Bowlby 1969). Although the inclusion of ELA predictors (e.g. physical abuse) significantly improved the model, the explained variance remained almost unchanged (R^2^ = 0.322 with CTQ physical abuse vs. R^2^ = 0.316 without CTQ), suggesting that although ELA contributes to individual differences in synchronization, its unique explanatory power within this model is modest.

To further examine the role of mental health status, we included participant group membership (patient vs. control) as a predictor in the models. The results showed that participants in the patient group exhibited significantly higher HF-HRV synchronization with the therapy horse compared to participants in the control group with an estimate of 0.26 (CI: 0.06–0.48, *p* <.05, as shown in Table 1). However, this group difference was not found for HF-HRV synchronization between the riding therapist and the participant. These findings indicate that participants with mental health challenges may exhibit increased physiological alignment specifically with therapy horses, compared to healthy controls. One possible explanation is that individuals undergoing psychotherapeutic treatment might engage differently with the therapy horse, potentially due to altered relational expectations or greater sensitivity to nonverbal, emotionally safe interactions. Given that a large proportion of participants in the patient group also reported elevated ELA, another possible interpretation is that prior relational trauma may contribute to this increased alignment with nonjudgmental, emotionally safe partners like therapy horses.

These observations raise an important hypothesis for future research: that PS may be moderated by ELA, either directly or indirectly via current mental health status. Future studies should explore whether higher ELA scores predict stronger participant-horse synchronization across both clinical and non-clinical samples, and whether this reflects a compensatory mechanism or a unique pathway of attachment and regulation in human-animal therapeutic contexts.

In summary, this research provides comprehensive insights into HF-HRV synchronization dynamics within the therapeutic triad of HAT sessions, emphasizing the significance of PS processes within and across dyads, along with potential associations with early life experiences.

However, the results of our study should be interpreted with several limitations in mind. First, with only four therapy horses, three riding therapists and only female participants aged between 18 and 45 years, our sample was relatively small and limited in its diversity. This limitation occurred primarily due to the difficulty in recruiting male participants who met the predetermined aged and weight criteria and were interested in HAT. In addition, we did not consider the phase of menstrual cycle, although there is evidence of significant hormonal influences on autonomic regulation and HRV fluctuations during the menstrual cycle (Brar et al. 2015; McKinley et al. 2009; Yildirir et al. 2002). This uncontrolled factor probably led to additional variability in our HRV measurements. Although therapy horses were randomly assigned to participants and therapists to control for selection bias, other potential confounding factors, such as individual differences in horse temperament, past training experiences, or prior relationships between horses and therapists, were not explicitly assessed or controlled in this study. Differences in horse temperament and previous experiences may influence the degree of PS (e.g. HRV patterns) during therapeutic interactions. Additionally, established bonds or familiarity between horses and therapists could also impact their physiological responsiveness and thus potentially confound results.

To overcome these limitations and improve precision, future research should systematically account for menstrual phase and recruit a more diverse sample that includes male participants, children and individuals over the age of 45. Additionally, future studies should systematically record and statistically control for potential confounding variables related to the therapy horses, such as individual differences in horse temperament, past training experiences and relationships with therapists, to further refine our understanding of HRV synchronization dynamics in HAT settings. Conducting longitudinal studies to investigate HRV synchronization patterns over extended periods of time would provide deeper insights into the stability and evolution of PS within HAT sessions, thereby significantly improving the generalizability and robustness of findings in this area of research.

HF-HRV synchronization is a complex, multifaceted phenomenon, and while this study focuses on HRV, other physiological and emotional variables that contribute to synchronization were not fully explored. Augmenting the analysis of HF-HRV synchronization with additional physiological measures could offer a more comprehensive view of emotional and physiological responses during HAT sessions. Additionally, investigating how individual participant characteristics, such as age, gender, and specific clinical conditions, correlate with HF-HRV synchronization could further enhance our understanding. As HF-HRV synchronization likely emerges from the interplay of multiple physiological, psychological, and contextual variables, future studies should aim to isolate these contributions through more mechanistic or experimentally controlled designs.

While this study provides valuable insights into the dynamics of HF-HRV synchronization within the therapeutic triad of HAT sessions, certain theoretical frameworks referenced in the discussion - such as attachment theory and therapist expectations - should be understood as guiding hypotheses rather than conclusive findings. These frameworks were not directly tested within the scope of this research but offer valuable perspectives that could inform future investigations. By framing these mechanisms as hypotheses, we encourage further research to explore their role in influencing physiological synchronization within HAT sessions.

While attachment theory provides a useful framework for interpreting our findings, alternative psychological mechanisms could also contribute to the observed PS patterns. For instance, mechanisms such as emotional contagion, empathic resonance, or attentional attunement could similarly explain heightened synchronization between therapy participants, horses and therapists. Emotional contagion – the autonomic imitation and synchronization of emotional states – could facilitate physiological alignment independently of bonding processes (Hatfield et al. 1994; Prochazkova and Kret 2017). Furthermore, empathic resonance (Decety and Jackson 2004; Singer and Lamm 2009) and attentional attunement (Gallese 2009) could enable therapists and horses to finely tune into the emotional states of participants and thereby influence PS.

However, these mechanisms alone cannot fully explain the lower synchronization observed in participants with ELA. While emotional contagion, empathic resonance, and attentional attunement could certainly contribute to PS, they do not adequately explain the impaired synchronization in individuals with ELA. ELA is often associated with disruptions in attachment and emotional regulation that may impair with the typical processes of emotional contagion and resonance, leading to reduced physiological alignment (Sigrist et al. 2021; Tell et al. 2018). The inability to effectively attune to the emotional states of others, a common consequence of ELA (Petrova et al. 2021), could therefore explain the lower synchronization observed in these individuals, again suggesting that attachment-related factors may be a crucial mechanism in this context.

The findings of this study offer valuable implications for clinical practice within HAT. The observed patterns of HF-HRV synchronization highlight the importance of promoting PS not only between participant and therapy horse but particularly within the dyad of riding therapist and horse. Given the association between therapist-horse PS and subsequent PS processes, therapists may consider proactively building general rapport and consistent interaction routines with their therapy horses prior to therapeutic sessions. Targeted trainings focused on improving the therapist’s awareness of physiological states and emotional regulation could further optimize these PS dynamics and potentially improve therapeutic outcomes. Furthermore, given the unique benefits for individuals with ELA, therapists can strategically use HAT as an intervention without the relational barriers that often occur in traditional therapeutic settings. Thus, integrating insights from PS into therapist training, horse selection, and session design could substantially enhance the effectiveness of HAT interventions.

In conclusion, this study not only advances the understanding of the physiological dynamics in HAT sessions but also opens avenues for refining therapeutic interventions by considering the nuanced influence of early life experiences on physiological synchronization, emphasizing the pivotal roles of the riding therapist, therapy horse, and their interplay in fostering positive therapeutic outcomes.

## Electronic supplementary material

Below is the link to the electronic supplementary material.

Supplementary Material 1

## Data Availability

The datasets generated and analyzed during the current study are accessible in the OSF-Repository (10.17605/OSF.IO/KNGBS). Additionally, the code utilized for the analysis is also available in the OSF-Repository (10.17605/OSF.IO/2W6EY).

## Declarations

All contributors of this study who have fulfilled the criteria for authorship required by springer nature journals have been included as authors.
