# Supplementary material for: The relationship of early life adversity and physiological synchrony within the therapeutic triad in horse-assisted therapy
Source: J Neural Transm (Vienna). 2025 May 27;132(9):1291–312. doi: 10.1007/s00702-025-02947-7 (PMC12535527; doi:10.1007/s00702-025-02947-7)
Supplement: Supplementary file 1 — Supplementary Material 1 [file 702_2025_2947_MOESM1_ESM.pdf]

The Relationship of Early Life Adversity and Physiological Synchrony within the Therapeutic Triad in Horse-Assisted Therapy

Supplementary section

Stella Wienhold[1,2,3], Larissa Bär[1,2], Zoe Ringleb[1,2,3], Victoria Zirpel[2], Annette Gomolla[2], Bernadette F. Denk[1,3], Nina Volkmer[1,3], Raphaela J. Gaertner[1], Elea S.C. Klink[1], and Jens C. Pruessner[1,3]

[1]University of Konstanz, Germany
[2]GREAT - German Research Center for Equine Assisted Therapy, Konstanz, Germany
[3]Centre for the Advanced Study of Collective Behaviour, Konstanz, Germany

Contents

Further Model Information **3**

Hypothesis 1: HRV synchronization between the participant and therapy horse varies depending on how synchronous the therapy horse and riding therapist are. 3

Hypothesis 2: HRV synchronization between participant and riding therapist varies depending on how synchronous the therapy horse and riding therapist are. 5

Hypothesis 3: HRV synchronization between the riding therapist and therapy horse is strongest when the therapist works with her favorite therapy horse. 7

Hypothesis 4a: Participants with pronounced ELA synchronize less with the therapy horse than with moderate to nonexistent ELA. 9

Hypothesis 4b: Participants with pronounced ELA synchronize less with the riding therapist than with moderate to nonexistent ELA. 9

List of Tables

S1 Comparison of Fit Indices for Models predicting HF-HRV Synchronization between Therapy Horse and Participant. 10

S2 Comparison of Fit Indices for Models predicting HF-HRV Synchronization between Riding Therapist and Participant. 12

S3 Comparison of Fit Indices for Models predicting HF-HRV Synchronization between Therapy Horse and Riding Therapist. 13

S4 Comparison of Fit Indices for Models predicting HF-HRV Synchronization between Therapy Horse and Participant, including CTQ Subscales. 14

S5 Comparison of Fit Indices for Models predicting HF-HRV Synchronization between Riding Therapist and Participant, including CTQ Subscales. 15

Further Model Information

Hypothesis 1: HRV synchronization between the participant and therapy horse varies depending on how synchronous the therapy horse and riding therapist are.

Hypothesis 1 was tested using a four-level model to analyze the HF-HRV synchronization between participants and therapy horses. The analysis began with a null model (Model 1) that lacked adjustments for predictor variables. Subsequent models were sequentially introduced, incorporating random intercepts, random slopes, covariates and the predictor variables Session, Minute, Interval, Frequency as well as interaction effects between the added predictor variables (Minute x Session, Minute x Interval, Interval x Session). The log-likelihood ratio was used to compare the overall model fit of the nested models. Once the best-fitting model was identified, the predictor variable HF-HRV Synchronization between Therapy Horse and Riding Therapist was integrated to evaluate its impact on HF-HRV synchronization between therapy horse and participant. The confirmation of the hypothesis rested on observing a significant main effect of the variable HF-HRV Synchronization between Therapy Horse and Riding Therapist within the multilevel model. The following models were gradually introduced:

- The incorporation of random intercepts to the Model significantly improved model fit (χ^2 = 179.18, $p < .01$).
- The addition of linear, quadratic or cubic fixed slopes for the time variable Session did not improve model fit significantly.
- Only the addition of linear random slopes for the time variable Session did improve model fit significantly, (χ^2 = 61.57, $p < .01$), while the quadratic and cubic random slopes for Session did not.
- The addition of linear and quadratic fixed slopes for the time variable Minute did improve model fit significantly (χ^2 = 235.59, $p < .01$, χ^2 = 65.88, $p < .01$), while the addition of cubic fixed slopes did not.

- Incorporating linear, quadratic and cubic random slopes for the time variable Minute did not improve model fit significantly, but the addition of the interaction between Session and Minute did ($\chi^2 = 7.19$, $p < .01$).

- The addition of linear fixed slopes for the time variable Interval did improve model fit significantly ($\chi^2 = 63.63$, $p < .01$). On the contrary, the addition of quadratic and cubic fixed slopes and the addition of linear random slopes for the variable Interval did not improve model fit significantly.

- The addition of the interactions between Minute and Interval and between Interval and Session did improve model fit significantly($\chi^2 = 93.24$, $p < .01$, $\chi^2 = 3.92$, $p < .05$).

- The addition of the variable Frequency referring to the frequency bands (UHF and LHF) significantly enhanced model fit ($\chi^2 = 582.00$, $p < .01$).

- Even though some of the explored covariates of the participant (Age, BMI, Medication, Psychotropic Medication, Experiences with Horses, Baseline HRV and Mental Health) did improve respective model fits ($\chi^2 = 0.14$, $p = .71$, $\chi^2 = 4.01$, $p < .05$; $\chi^2 = 0.01$, $p = .91$; $\chi^2 = 0.05$, $p = .82$; $\chi^2 = 0.41$, $p = .52$, $\chi^2 = 7.87$, $p < .01$, $\chi^2 = 6.52$, $p < .05$,), only BMI, Baseline HRV and Mental Health reached statistical significance as predictors in the respective model. Therefore, these variables were included as covariates in the final model for hypothesis testing.

- Adding the variable HF-HRV Synchronization between Therapy Horse and Riding Therapist to the model improved model fit significantly ($\chi^2 = 1770.83$, $p < .01$), confirming Hypothesis 1.

Hypothesis 2: HRV synchronization between participant and riding therapist varies depending on how synchronous the therapy horse and riding therapist are.

Hypothesis 2 was evaluated using a parallel analytical approach outlined in Hypothesis 1. Commencing with the establishment of the null model, the focus was on predicting the HF-HRV synchronization between the riding therapist and the participant. The subsequent steps and the comparison of model fits, followed those of Hypothesis 1. The independent variable HF-HRV Synchronization between Therapy Horse and Riding Therapist was incorporated as a predictor variable to examine its influence on the HF-HRV synchronization between the riding therapist and the participant. The hypothesis was confirmed, as a significant main effect of the variable HF-HRV Synchronization between Therapy Horse and Riding Therapist was observed within the multilevel model.

- The incorporation of random intercepts to the Model significantly improved model fit ($\chi^2 = 220.13$, $p < .01$).
- The addition of linear, quadratic and cubic fixed slopes for the time variable Session did not improve model fit significantly.
- Only the addition of linear random slopes for the time variable Session did improve model fit significantly, ($\chi^2 = 46.79$, $p < .01$), while the quadratic and cubic random slopes for Session did not.
- The addition of linear and quadratic fixed slopes for the time variable Minute did improve model fit significantly ($\chi^2 = 178.98$, $p < .01$, $\chi^2 = 20.43$, $p < .01$), while the addition of cubic fixed slopes did not.
- Incorporating linear, quadratic and cubic random slopes for the time variable Minute did not improve model fit significantly, but the addition of the interaction between Session and Minute did ($\chi^2 = 4.10$, $p < .05$).
- The addition of linear, quadratic and cubic fixed slopes for the time variable Interval did not improve model fit significantly.

- The addition of the interactions between Minute and Interval did improve model fit significantly ($\chi^2 = 4.81$, $p < .05$), as well as the interaction between Interval and Session did ($\chi^2 = 5.29$, $p < .05$).

- The addition of the variable Frequency referring to the frequency bands (UHF and LHF) significantly enhanced model fit ($\chi^2 = 1147.95$, $p < .01$).

- Of all participant and riding therapist covariates examined, only the riding therapist's age and baseline HRV significantly improved model fit ($\chi^2 = 4.20$, $p < .05$, $\chi^2 = 36.93$, $p < .01$), and were thus the only covariates included in the final model for hypothesis testing.

- Adding the variable HF-HRV Synchronization between Therapy Horse and Riding Therapist to the model improved model fit significantly ($\chi^2 = 757.87$, $p < .01$), confirming Hypothesis 2.

Hypothesis 3: HRV synchronization between the riding therapist and therapy horse is strongest when the therapist works with her favorite therapy horse.

Hypothesis 3 was tested was evaluated using a parallel analytical approach outlined in Hypothesis 1 and 2. The critical distinction was that the dependent variable was the HF-HRV synchronization between the therapy horse and the riding therapist. The process began with the establishment of the null model (Model 3). Subsequently, we proceeded through the steps of incorporating random intercepts, introducing predictor variables, and assessing interaction effects, mirroring the process outlined for Hypothesis 1 and 2. Once the best-fitting model was identified, the impact of the riding therapist-therapy horse relationship was evaluated by adding the independent variables Therapy Horse and Favorite Therapy Horse. The hypothesis was confirmed when a significant main effect of the variable Therapy Horse and Favorite Therapy Horse was found within the multilevel model. The following models were gradually introduced:

- The incorporation of random intercepts to the Model significantly improved model fit ($\chi^2 = 54.44$, $p < .01$).
- The addition of linear, quadratic or cubic fixed slopes for the time variable Session did not improve model fit significantly, neither did the addition of linear, quadratic or cubic random slopes.
- The addition of linear and quadratic fixed slopes for the time variable Minute did improve model fit significantly ($\chi^2 = 148.10$, $p < .01$, $\chi^2 = 51.29$, $p < .01$), while the addition of cubic fixed slopes did not.
- Incorporating linear, quadratic and cubic random slopes for the time variable Minute did not improve model fit significantly, neither did the addition of the interaction between Session and Minute.
- The addition of linear fixed slopes for the time variable Interval did improve model fit significantly ($\chi^2 = 19.97$, $p < .01$). On the contrary, the addition of quadratic and cubic fixed slopes and the addition of linear random slopes for the variable Interval did not improve model fit significantly.

- The addition of the interaction between Minute and Interval did improve model fit significantly (χ^2 = 57.93, $p < .01$), whereas that of the interaction between Interval and Session did not (χ^2 = 1.29, $p = .25$).
- Adding the variable Frequency for respective frequency bands (UHF and LHF) significantly enhanced model fit (χ^2 = 1176.05, $p < .01$).
- Even though all of the explored covariates (Age, BMI, medication and Baseline HRV) of the riding therapist improved model fit at first (χ^2 = 5.91, $p < .05$; χ^2 = 8.45, $p < .01$; χ^2 = 8.73, $p < .01$; χ^2 = 37.85, $p < .01$), only the covariate Baseline HRV did reach statistical significance in the respective model. This suggests that the other covariates contribute to explaining variance in the dependent variable, although their individual effect was not statistically significant. Therefore, only Baseline HRV of the riding therapist was included in the final model for hypothesis testing.
- None of the explored CTQ predictor variables (of the riding therapist) Totalscore, Emotional Abuse, Emotional Neglect, Physical Abuse, Physical Neglect and Sexual Abuse did improve respective model fits.
- Adding the variable Therapy Horse and Favorite Therapy Horse to the model significantly enhanced respective model fit (χ^2 = 31.21, $p < .01$, χ^2 = 4.28, $p < .05$).

Hypothesis 4a: Participants with pronounced ELA synchronize less with the therapy horse than with moderate to nonexistent ELA.

The reference model used to test Hypothesis 4a was the established Model 1, which had already been employed in the process of examining Hypothesis 1. Early Life Adversity was introduced using the CTQ subscales Emotional Abuse, Emotional Neglect, Physical Abuse, Physical Neglect, and Sexual Abuse as predictors to investigate their impact on HF-HRV synchronization. This approach was motivated by the recognition that different forms of ELA can yield distinct effects on both psychological and physiological responses. By examining the respective subscales, we aimed to gain a nuanced understanding of how each type of adversity may uniquely influence HF-HRV synchronization. The hypothesis was validated as a significant main effect of the CTQ predictor variables was found within the multilevel model.

Hypothesis 4b: Participants with pronounced ELA synchronize less with the riding therapist than with moderate to nonexistent ELA.

To test hypothesis 4b, the procedure was similar to the analysis used to test hypothesis 4a, with the only difference that the predicted variable being HF-HRV synchronization between the riding therapist and participant and therefore the reference model being Model 2. The hypothesis was supported as a significant main effect of CTQ predictor variables was found within the multilevel model. In addition, the CTQ predictor variables of the riding therapist were included in the respective model to investigate whether the riding therapist's ELA also had an effect on the HF-HRV synchronization between therapist and participant, but none of these variables significantly improved the model fit.

Table S1

Comparison of Fit Indices for Models predicting HF-HRV Synchronization between Therapy Horse and Participant.

Model	χ^2			AIC	BIC
	Value	df	p		
Nullmodel (M1)<br>*XWP_HP ∼ 1*				15586.24	15599.45
M1 + random = ∼ 1| P<br>*Nullmodel M2 with random intercepts*	179.18	1	< **.01**	15409.06	15428.88
M1 + .∼. + random = ∼ Session|P<br>*Model with random effect for Session (linear)*	61.57	1	< **.01**	15351.49	15384.53
M1 + .∼. + Minute<br>*Model with fixed effect for Minute (linear)*	235.59	1	< **.01**	15117.90	15157.55
M1 + .∼.+ Minute^2<br>*Model with fixed effect for Minute (quadratic)*	65.88	1	< **.01**	15054.02	15100.27
M1 + .∼. + Session:Minute<br>*Model with interaction for Session and Minute*	7.19	1	< **.01**	15048.83	15101.69
M1 + .∼. + Interval<br>*Model with fixed effect for Interval (linear)*	63.63	1	< **.01**	14987.20	15046.67
M1 + .∼. + Minute:Interval<br>*Model with interaction for Minute and Interval*	93.24	1	< **.01**	14895.96	14962.03
M1 + .∼. + Session:Interval<br>*Model with interaction for Session and Interval*	3.92	1	< **.05**	14894.04	14966.72
M1 + .∼. + Frequency<br>*Model with predictor Frequency*	582.00	1	< **.01**	14314.03	14393.32
M1 + .∼. + BMI (P)<br>*Model with predictor BMI of participant*	4.01	1	< **.05**	14312.03	14397.92
M1 + .∼. + Baseline HRV (P)<br>*Model with predictor baseline HRV of participant*	7.87	1	< **.01**	14306.16	14398.66
M1 + .∼. + group (P)<br>*Model with predictor group (EG or CG) of participant*	6.52	1	< **.05**	14301.64	14400.75
M1 + .∼. + XWP of TH and RT<br>*Model with predictor HF-HRV synchronization of horse and therapist*	1770.83	3	< **.01**	12532.81	12638.53

Note. The table presents fit indices for various models predicting HF-HRV synchronization between therapy horse and participant. The fit indices include chi-square value (χ^2), degrees of freedom (df), and p-value (p) for the chi-square test in reference to the best-fitted model before each iteration, as well as the Akaike Information Criterion (AIC) and Bayesian Information Criterion (BIC).

Table S2

Comparison of Fit Indices for Models predicting HF-HRV Synchronization between Riding Therapist and Participant.

Model	χ^2			AIC	BIC
	Value	df	p		
Nullmodel (M2)<br>*XWP_HT ~ 1*				15609.97	15623.19
M2 + random = ~ 1| P<br>*Nullmodel M2 with random intercepts*	220.13	1	< **.01**	15391.84	15411.66
M2 + .~. + random = ~ Session|P<br>*Model with random effect for Session (linear)*	46.79	1	< **.01**	15349.05	15382.08
M2 + .~. + Minute<br>*Model with fixed effect for Minute (linear)*	178.98	1	< **.01**	15172.07	15211.71
M2 + .~.+ Minute^2<br>*Model with fixed effect for Minute (quadratic)*	20.43	1	< **.01**	15153.63	15199.89
M2 + .~.+ Session:Minute<br>*Model with interaction for Session and Minute*	4.10	1	< **.01**	15151.54	15204.40
M2 + .~. + Minute:Interval<br>*Model with interaction for Minute and Interval*	4.81	1	< **.05**	15148.73	15208.20
M2 + .~. + Session:Interval<br>*Model with interaction for Session and Interval*	5.29	1	< **.05**	15145.44	15211.51
M2 + .~. + Frequency<br>*Model with predictor Frequency*	1147.95	1	< **.01**	13999.49	14072.17
M2 + .~. + age (RT)<br>*Model with predictor age of RT*	4.20	1	< **.01**	13997.29	14076.58
M2 + .~. + Baseline HRV (RT)<br>*Model with predictor Baseline HRV of RT*	36.93	1	< **.01**	13962.36	14048.26
M2 + .~. + XWP of TH and RT<br>*Model with predictor HF-HRV synchronization of horse and therapist*	757.87	3	< **.01**	13206.49	13298.99

Note. The table presents fit indices for various models predicting HF-HRV synchronization between riding therapist and participant. The fit indices include chi-square value (χ^2), degrees of freedom (df), and p-value (p) for the chi-square test in reference to the best-fit model before each iteration, as well as the Akaike Information Criterion (AIC) and Bayesian Information Criterion (BIC).

Table S3

Comparison of Fit Indices for Models predicting HF-HRV Synchronization between Therapy Horse and Riding Therapist.

Model		χ^2		AIC	BIC
	Value	df	p		
Nullmodel (M3)				15592.75	15605.96
XWP ∼ 1					
M3 + random = ∼ 1|RT	54.44	1	< **.01**	15540.31	15560.13
Nullmodel M1 with random intercepts					
M3 + .∼. + Minute	148.10	1	< **.01**	15394.21	15420.64
Model with fixed effect for Minute (linear)					
M3 + .∼.+ Minute^2	51.29	1	< **.01**	15344.92	15377.96
Model with fixed effect for Minute (quadratic)					
M3 + .∼.+ Interval	19.97	1	< **.01**	15326.95	15366.60
Model with fixed effect for Interval (linear)					
M3 + .∼. + Minute:Interval	57.93	1	< **.01**	15271.03	15317.28
Model with interaction for Minute and Interval					
M3 + .∼. + Frequency	1176.05	1	< **.01**	14096.98	14149.84
Model with predictor Frequency					
M3 + .∼. + Baseline HRV (RT)	39.10	1	< **.01**	14059.88	14119.34
Model with predictor Baseline HRV of riding therapist					
M3 + .∼. + Therapy Horse	31.21	3	< **.01**	14034.67	14113.96
Model with predictor Therapy Horse					
M3 + .∼. + Favorite Therapy Horse	4.28	1	< **.01**	14032.39	14118.29
Model with predictor Favorite Therapy Horse					

Note. The table presents fit indices for various models predicting HF-HRV synchronization between therapy horse and riding therapist. The fit indices include chi-square value (χ^2), degrees of freedom (df), and p-value (p) for the chi-square test in reference to the best-fit model before each iteration, as well as the Akaike Information Criterion (AIC) and Bayesian Information Criterion (BIC).

Table S4

Comparison of Fit Indices for Models predicting HF-HRV Synchronization between Therapy Horse and Participant, including CTQ Subscales.

Model		χ^2		AIC	BIC
	Value	df	p		
M1				12532.81	12638.53
Model with predictor HF-HRV synchronization of horse and therapist					
M1 + .∼. + CTQ Total	4.51	1	< **.05**	12530.31	12642.63
Model with predictor CTQ Totalscore					
M1 + .∼. + CTQ Emotional Abuse	4.99	1	< **.05**	12529.82	12642.15
Model with predictor CTQ Emotional Abuse					
M1 + .∼. + CTQ Emotional Neglect	4.98	1	< **.05**	12529.84	12642.16
Model with predictor CTQ Emotional Neglect					
M1 + .∼. + CTQ Physical Abuse	1.23	1	.21	12533.59	12645.91
Model with predictor CTQ Physical Abuse					
M1 + .∼. + CTQ Physical Neglect	3.88	1	< **.05**	12530.93	12643.25
Model with predictor CTQ Physical Neglect					
M1 + .∼. + CTQ Sexual Abuse	0.47	1	.49	12534.34	12646.67
Model with predictor CTQ Sexual Abuse					

Note. The table presents fit indices for various models predicting HF-HRV synchronization between therapy horse and participant. The fit indices include chi-square value (χ^2), degrees of freedom (df), and p-value (p) for the chi-square test in reference to the base model M2, as well as the Akaike Information Criterion (AIC) and Bayesian Information Criterion (BIC).

Table S5

Comparison of Fit Indices for Models predicting HF-HRV Synchronization between Riding Therapist and Participant, including CTQ Subscales.

Model	χ^2			AIC	BIC
	Value	df	p		
M2 + .∼. + XWP of TH and RT				13206.49	13298.99
Model with predictor HF-HRV synchronization of horse and therapist					
M2 + .∼. + CTQ Total	5.69	1	< **.05**	13202.80	13301.91
Model with predictor CTQ Totalscore					
M2 + .∼. + CTQ Emotional Abuse	4.64	1	< **.01**	13203.85	13302.96
Model with predictor CTQ Emotional Abuse					
M2 + .∼. + CTQ Emotional Neglect	6.67	1	< **.01**	13201.82	13300.93
Model with predictor CTQ Emotional Neglect					
M2 + .∼. + CTQ Physical Abuse	4.72	1	< **.05**	13203.76	13302.88
Model with predictor CTQ Physical Abuse					
M2 + .∼. + CTQ Physical Neglect	1.30	1	.26	13207.19	13306.30
Model with predictor CTQ Physical Neglect					
M2 + .∼. + CTQ Sexual Abuse	2.71	1	.10	13205.78	13304.89
Model with predictor CTQ Sexual Abuse					

Note. The table presents fit indices for various models predicting HF-HRV synchronization between riding therapist and participant. The fit indices include chi-square value (χ^2), degrees of freedom (df), and p-value (p) for the chi-square test in reference to the base model M3, as well as the Akaike Information Criterion (AIC) and Bayesian Information Criterion (BIC).
